# A Review on the Recent Advancements of Ni-Based Sulfides and Mixed Sulfides for Supercapacitors and Electrocatalysis (Oxygen Evolution Reaction)

**DOI:** 10.3390/molecules30132877

**Published:** 2025-07-07

**Authors:** Ganesh Dhakal, Sumanta Sahoo, Krishna Prasad Sharma, Guang-Lin Zhao

**Affiliations:** 1 Department of Physics and Nano Materials Laboratory, Southern University and A&M College, Baton Rouge, LA 70813, USA; 2 School of Chemical Engineering, Yeungnam University, 280 Daehak-ro, Gyeongsan 38541, Republic of Korea

**Keywords:** nickel sulfide, nickel mixed sulfide, supercapacitor, oxygen evolution reaction

## Abstract

Energy storage and conversion units have been considered the backbone of modern energy science and technology. In recent years, the Ni-based sulfides (NS) and mixed sulfides (NMS) have been significantly utilized as promising electrodes for various energy-related applications. This article summarizes the recent progress of NS and NMS materials in the fields of energy storage (supercapacitors) and conversion (oxygen evolution reactions). The synthetic approaches have been thoroughly discussed. A brief overview of the electrochemical performance of these materials as the electrodes for energy storage and conversion is systematically represented in the article. For such applications, these materials are frequently combined with other advanced materials, such as metal oxides, metal sulfides, and carbonaceous materials. The article ends with the existing challenges and future research directions in these research fields.

## 1. Introduction

Electrochemical energy storage and conversion systems have been considered as the pioneer technology of the current century. The storage of energy and its conversion from one form to another through electrochemical processes has drawn considerable research attention because of their easy processability, diverse range of applications, and huge demand for future technology. In this aspect, the energy storage devices, like secondary batteries and supercapacitors (SCs), have been considered as the key devices [1,2,3,4,5]. On the other hand, the oxygen evolution reaction (OER) is assigned as the vital component for constructing the energy conversion [6,7,8,9,10]. Such components not only advance future technology but also promote renewable energy resources, which further leads to the formation of a green and sustainable future.

Metal oxides and mixed metal oxides have been synthesized through diverse synthetic routes for constructing energy electrodes. Specifically, the transition metal oxides/mixed oxides demonstrated superior supercapacitive performance over their other metal counterparts [11,12,13]. Significant research has also been conducted in the case of metal sulfides and mixed metal sulfides. These materials display higher electrochemical characteristics over their oxide counterparts because of richer surface chemistry, enhanced conductive nature, and higher electronic properties. Compared to other transition metals, Ni has the advantages of richer redox activity, tunable structure and composition, better stability, lower cost, and higher abundance. Among transition metals, Ni is considered a potential component for fabricating energy electrodes due to its favorable electrochemical activity. In this aspect, the Ni-based metal oxide (NiO) and mixed oxide (NiCo_2_O_4_) have been widely investigated [14,15,16]. In the last few years, Ni-based sulfides (NS) and mixed sulfides (NMS) have been extensively investigated for developing electrodes for energy storage and conversion [17,18,19,20,21]. In order to improve the performance, such materials have further been combined with other metal sulfides/mixed sulfides, metal oxides/mixed oxides, carbon materials, MXenes, conducting polymers, etc. [22,23,24].

**Figure 1:**
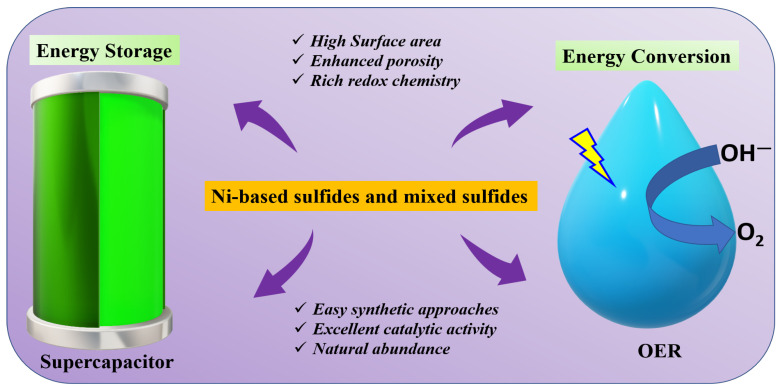
Schematics of the aim of the current review article, highlighting the advantages of Ni-based sulfide and mixed sulfide materials.

The current article reviews the most recent advancements of NS and NMS in the field of energy storage (SC) and conversion (OER). The research investigations of these materials in the last 5 years (2020–2025) are thoroughly discussed. The article starts with a brief introduction to the above-mentioned energy storage and conversion applications. The synthetic approaches are further summarized. Lastly, the article ends with the conclusion and future prospects. Figure 1 represents the prime aim of the review article. As shown, the NS and NMS materials have been frequently utilized for energy-related applications because of their high surface area, enhanced porosity, rich redox activity, easy synthetic approaches, high catalytic activity, and natural abundance.

## 2. Brief Discussion on Energy Storage and Conversion

Energy storage and conversion of one form to another have become crucial for the development of a sustainable world. Specifically, renewable energy resources have been considered as valuable assets of modern science and technology. Among the energy storage devices, SCs are considered promising because of their fast charge–discharge ability, longer cycle life, and easy fabrication processes. According to the charge storage mechanism, such devices are classified into two classes: pseudocapacitive (store charge through Faradaic reactions) and electric-double-layer capacitors (EDLC) (store charge through a non-Faradaic fashion) [1,25,26,27]. A great deal of research has been conducted on the synthesis of the SC electrodes. The carbon-based materials generally store charges through the EDLC charge storage mechanism [28,29,30]. On the other hand, the metal oxides, metal chalcogenides, conductive polymers, etc., follow the pseudocapacitive charge storage mechanism [31,32,33,34]. It is important to note that the electrochemical performance of SC electrodes depends on several factors, including the surface area, porosity, conductivity, and crystallinity of the electrode materials. On the other hand, the electrolytes also play a significant role in the performance of the SCs. The concentration, ionic conductivity, and nature of the electrolytes expressively influence the charge–storage ability of the electrodes. For example, a previous report demonstrated the increment of capacitance of an SC electrode based on NiCo_2_O_4_ by enhancing the concentration of KOH electrolyte [35]. The combination of pseudocapacitive and EDLC-type materials produces the hybrid SC electrodes, which are nothing but composite-type materials. These materials are constructed to utilize the advantages of both the pseudocapacitor and EDLC. The SC devices are constructed by combining the positive and negative electrode materials. In general, the carbon-based materials, like activated carbon, graphene, etc., are being utilized as the negative electrodes. The current research trend in SC has moved towards the exploration of sustainable resources for the fabrication of electrodes. In this context, the biomass-derived carbon materials have been considered as the pioneer materials for SCs.

Electrochemical water splitting (EWS) is an efficient method for producing hydrogen and oxygen by breaking down water molecules through the hydrogen evolution reaction (HER) and OER [6,7,8,10,36]. Although the theoretical cell voltage essential for water splitting is 1.23 V, in practice, a higher voltage, known as overpotential (OP), is needed to drive the reaction efficiently. To reduce this OP and improve overall efficiency, effective and affordable electrocatalysts are essential. Precious metal-based catalysts such as platinum (Pt) and ruthenium oxide (RuO_2_) have been used for HER and OER and have demonstrated excellent catalytic performance. However, their high cost, limited availability, and environmental concerns restrict their large-scale and long-term use. Therefore, there is a growing need to develop low-cost, abundant, and green alternative electrocatalysts to make water splitting. The selection of the electrocatalysts highly affects the water splitting performance. Additionally, the stability and efficiency of these materials also influence the overall catalytic activity. Extensive research has been conducted on the development of electrocatalysts for water splitting, focusing on a wide range of materials, including transition metal oxides, sulfides, phosphides, nitrides, and carbon-based compounds [37,38]. Among these, transition metal sulfide-based electrocatalysts have gained significant attention due to their excellent catalytic activity, natural abundance, cost-effectiveness, and high surface area. These properties make them promising alternatives to traditional Pt-based catalysts, which, although highly efficient, are expensive and scarce.

## 3. Synthetic Approaches

**Figure 2:**
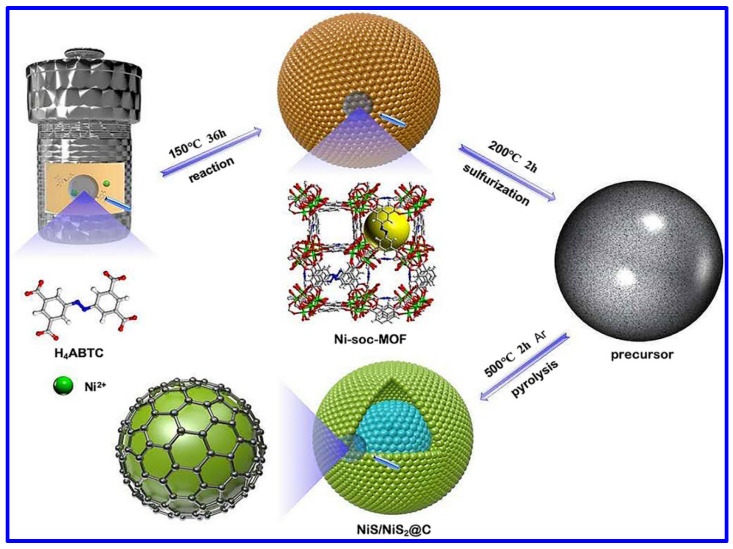
Schematics of the synthetic approach of NiS/NiS_2_@carbon composite [41].

The NS and NMS materials have been extensively synthesized through versatile synthetic approaches. Among them, the most common synthetic approach is the hydrothermal process. It is important to note that such a specific synthetic route has not only been employed for synthesizing NS and NMS-based powder materials but also explored to grow such materials in various conductive substrates; for example, Ni foam (NF). In this aspect, Yan et al. reported the hydrothermal synthesis of NiS nanosheets on NF [39]. In a typical process, the Ni-based salt, Ni(NO_3_)_2_, along with urea and NF, was hydrothermally treated at 180 °C for 18 h at a heating rate of 3 °C/min. In the next step, the coated foam was further hydrothermally heated at 120 °C for 3 h along with Na_2_S, which was used as the S-source. The morphological analysis confirmed the growth of NiS nanosheets. In another work, β-NiS was hydrothermally synthesized using Ni(CH_3_COO)_2_·4H_2_O salt [40]. However, in this case, the reaction temperature was set at 150 °C for a reaction time of 12 h for the synthesis of metal sulfide nanoparticles. The hydrothermal synthetic approaches associated with the post-heat treatment are reported for synthesizing various NS-based materials with diverse morphologies and related composites. The metal–organic framework (MOF)-derived NS materials have also been synthesized through this approach. In this aspect, Wang et al. demonstrated the hydrothermal-assisted synthesis of an MOF-derived NiS/NiS_2_@carbon composite, as shown in Figure 2 [41]. For the MOF synthesis, 3,3′,5,5′-azobenzenetetracarboxylic acid (H_4_ABTC) was used, and the MOF-derived NS material was synthesized through pyrolysis at 500 °C in an Ar atmosphere. Hydrothermal synthesis was further employed for the synthesis of the composite based on Cu_2_S, NiS, Ni_3_S_4_, and MnO_2_ [42]. In another work, P-doped Co_3_S_4_ was combined with Ni_3_S_4_ on NF through a two-step hydrothermal approach, where NaH_2_PO_2_ was utilized as the doping agent in the phosphating method [43].

**Figure 3:**
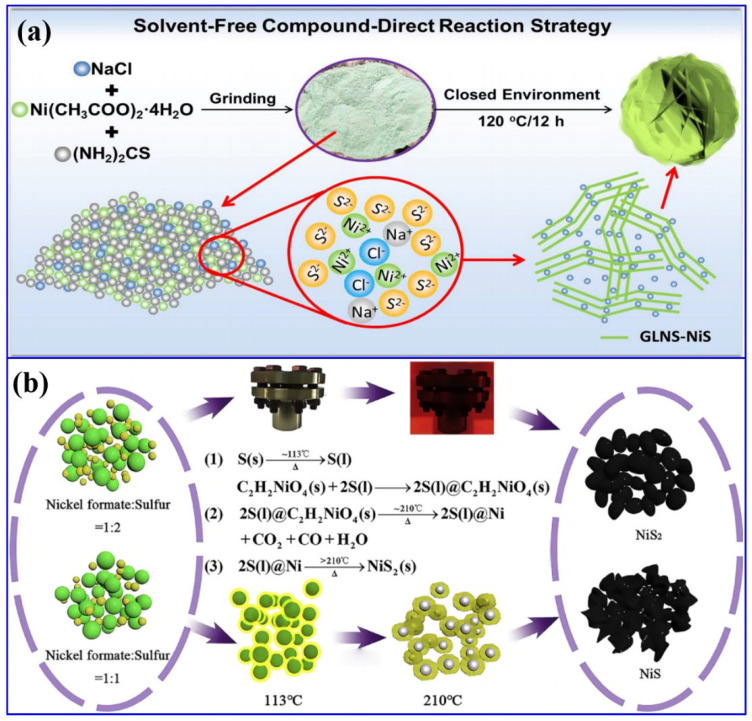
Solid-state synthesis of NS-based materials: (**a**) schematics of the solvent-free synthetic approach of graphene-like ultra-thin NiS nanosheets (GLNS-NiS) [44]; (**b**) schematics and the related reactions for the synthesis of NiS and NiS_2_ [45].

Apart from hydrothermal, the solvent-free direct synthesis of NiS with nanosheet morphology was reported [44]. In this process, Ni-containing salt, Ni (CH_3_COO)_2_·4H_2_O, (NH_2_)_2_CS, and NaCl were ground and further heat-treated at 120 °C for 12 h in a blast oven, resulting in the formation of graphene-like ultra-thin NiS nanosheets (Figure 3a). Herein, the addition of NaCl regulated the growth process and prevented the thickness of NiS nanosheets. The associated reactions are provided below:

Ni(CH_3_COO)_2_·4H_2_O → Ni(CH_3_COO)_2_ + 4H_2_O (1)

(NH_2_)_2_CS + 2H_2_O → CO_2_ + 2NH_3_ + H_2_S (2)

Ni(CH_3_COO)_2_ + H_2_S → NiS + 2CH_3_COOH (3)

Apart from hydrothermal, the solvent-free direct synthesis of NiS with nanosheet morphology was reported [44]. In this process, Ni-containing salt, Ni (CH_3_COO)_2_·4H_2_O, (NH_2_)_2_CS, and NaCl were ground and further heat-treated at 120 °C for 12 h in a blast oven, resulting in the formation of graphene-like ultra-thin NiS nanosheets (Figure 3a). Herein, the addition of NaCl regulated the growth process and prevented the thickness of NiS nanosheets. The associated reactions are provided below:

Ni(CH_3_COO)_2_·4H_2_O → Ni(CH_3_COO)_2_ + 4H_2_O (1)

(NH_2_)_2_CS + 2H_2_O → CO_2_ + 2NH_3_ + H_2_S (2)

Ni(CH_3_COO)_2_ + H_2_S → NiS + 2CH_3_COOH (3)

The formation of the final product after the heat treatment can be visualized with the naked eye through the transformation of the color from green (after grinding) to black. In another work, another solid-state synthetic approach was reported for the synthesis of both NiS and NiS_2_ [45]. In this process, initially Ni formate and sulfur powder of the desired quantity were ground and calcined at a temperature of 440 °C for a reaction time of 30 min to synthesize the NiS_2_ nanoparticles. The optimization of temperature resulted in the formation of NiS (Figure 3b). Compared to other conventional approaches, these solid-state processes have the advantages of the absence of hazardous chemicals and an easy synthetic protocol. In another work, a combination of the dealloying method and ion-exchange process resulted in the formation of NiS/NiO nanoparticles [46]. In this process, initially, the Al_91_Ni_9_ alloy was dealloyed, followed by a calcination process to form NiO. The further sulfuration process yielded a composite of NiO and NiS. The solvent-free approaches were further employed in another work to synthesize α-NiS, β-NiS, and a combination of both types of NS materials [47]. In another work, a single-step electrodeposition process has been employed to synthesize a ternary composite based on Co_9_S_8_, NiS_2_, and Cu_2_S on NF [48]. In another work, a solvothermal synthetic route was conducted to synthesize Mg-MOF and Ni-MOF using terephthalic acid as the MOF precursor. Such MOFs have been utilized as precursors to form NS-based composites. In the next step, these precursors were hydrothermally treated along with Na_2_S as the sulfur source to form the composite of Mg(OH)_2_ and Ni_3_S_4_ [49]. The above-mentioned discussion concludes that the NS materials have been synthesized and subsequently combined with other potential functional materials for their utilization in the field of energy storage and conversion.

**Figure 4:**
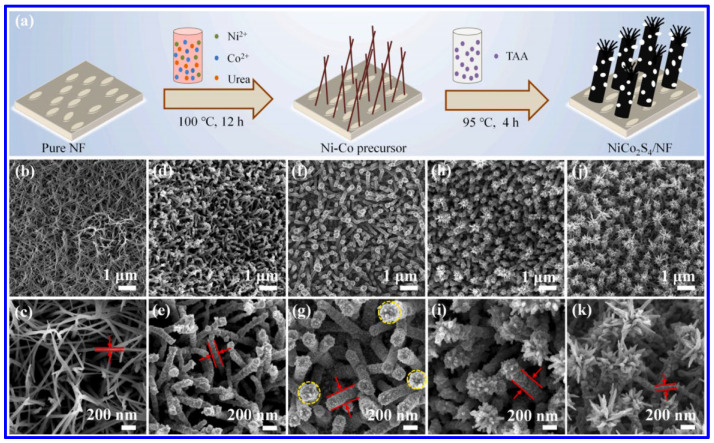
(**a**) Schematics of the hydrothermal synthesis of NiCo_2_S_4_ on NF; the SEM images of various NiCo_2_S_4_-coated NF after hydrothermal treatment of different time intervals: (**b**,**c**) Ni-Co precursor (after first hydrothermal treatment); (**d**,**e**) NiCoS-2 (after second hydrothermal reaction time of 2 h); (**f**,**g**) NiCoS-3 (after second hydrothermal reaction time of 3 h); (**h**,**i**) NiCoS-4 (after second hydrothermal reaction time of 4 h); (**j**,**k**) NiCoS-5 (after second hydrothermal reaction time of 5 h) [50].

Similar to the NS materials, NMS materials have also been frequently synthesized through the hydrothermal approach. Among the MNS materials, NiCo_2_S_4_ is the most-studied one for SC applications. In this aspect, Wang et al. demonstrated the hydrothermal synthesis of NiCo_2_S_4_ nanorods on NF (Figure 4a) [50]. In this approach, initially, the Ni and Co salts were hydrothermally treated along with urea at a temperature of 100 °C for 12 h to form the network-like structures of Ni-Co precursor. In the next step, the hydrothermal treatment (95 °C, 4 h) of the coated NF, along with thioacetamide (acting as the Sulfur source), resulted in the formation of the nanorods of NiCo_2_S_4_. The corresponding morphological images represent the formation of the nanorod arrays of the MNS material on the NF, as shown in the SEM images in Figure 4b–k. After the first hydrothermal treatment, the interconnected networks of the Ni-CO precursors were formed (Figure 4b,c). The nanorod arrays of NiCo_2_S_4_ were formed after the second hydrothermal treatment for 2 h (Figure 4d,e). With an increase in the reaction time of 3 h, the diameter of the nanorods has been enhanced and, further, the tip of the rods became rougher (Figure 4f,g). At the reaction time of 4 h, the flower-like structure is visible (Figure 4h,i). Further increments in reaction time to 5 h resulted in the full blooming of the flowers (Figure 4j,k). During the first hydrothermal treatment, the urea acted as the hydrolyzing agent to produce the OH^−^ and CO_3_^2−^ ions, which further reacted with the Ni^2+^ and Co^2+^ ions to form the Ni-Co precursor on NF. Further hydrothermal treatment with the S agent resulted in the formation of MNS materials through further reaction and an ion-exchange process. The sulfurization of the MNS material is also reported with a single-step hydrothermal process. In order to control the shape, surfactants have also been utilized. In this context, a recent report suggested a hydrothermal treatment of the nitrate salts of Ni and Co, urea, and Na_2_S·9H_2_O, along with the hexamethylenetetramine (HMTA), which acted as the surfactant to form the nanosheets of NiCo_2_S_4_ [51]. Such hydrothermal processes have been further utilized to prepare the composite SC electrodes based on NiCo_2_S_4_, like NiCo_2_S_4_/functionalized multi-walled carbon nanotube (MWCNT) [52], NiCo_2_S_4_/N, S co-doped graphene [53], NiCo_2_S_4_/MoS_2_ [54], etc. In a recent work, a microwave-assisted approach has been reported for the synthesis of the composite based on NiCo_2_S_4_ and reduced graphene oxide (RGO) [55]. In this process, initially, the graphite oxide solution was mixed with the ethylene glycol, nitrate salts of Co and Ni, and CS_2_. In the next step, the solution was heated for 15 min at a temperature of 150 °C in a microwave reactor (1000 W) to form the composite. Compared to the conventional approaches, this microwave-assisted approach has the advantage of a shorter reaction time.

**Figure 5:**
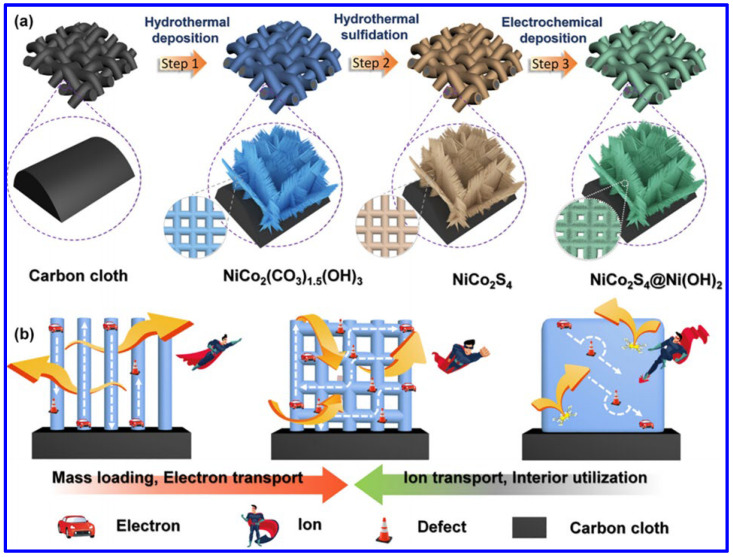
(**a**) Schematics of NiCo_2_S_4_/Ni(OH)_2_ meshes on carbon cloth, and (**b**) the advantages of forming such a composite in the field of SC [56].

Apart from the NF, the NiCo_2_S_4_ was also synthesized on other substrates. For example, Chen et al. demonstrated the formation of NiCo_2_S_4_ meshes through a two-step hydrothermal process and then combined them with Ni(OH)_2_ through electrochemical deposition (Figure 5a) [56]. The unique combination of the 2D nanosheets (NiCo_2_S_4_) and 1D nanowires (Ni(OH)_2_) has significant advantages for the fabrication of binder-free SC electrodes in terms of higher mass loading, easy electron and ion transport, and the prominent usage of interior active materials (Figure 5b). As a result, the electrode displayed higher areal capacity. The probable electrochemical reactions during the first hydrothermal process of forming the Ni-Co precursor on carbon cloths are provided below:

NH_4_F → NH_4_^+^ + F^−^ (4)

Co(NH_2_)_2_ + H_2_O → 2NH_3_ + CO_2_ (5)

NH_3_ + H_2_O → NH_4_^+^ + OH^−^ (6)

Ni^2+^ + 2Co^2+^ + 6OH^−^ → NiCo_2_(OH)_6_ (7)

CO_2_ + H_2_O → CO_3_^2−^ + 2H^+^ (8)

NiCo_2_(OH)_6_ + 1.5CO_3_^2−^ + 0.1H_2_O → NiCo_2_(CO_3_)1.5(OH)_3_ · 0.11H_2_O + 3OH^−^ (9)

Apart from NiCo_2_S_4_, other NMS materials have also been synthesized through different synthetic approaches. Min et al. reported the two-step hydrothermal synthesis of the FeNi_2_-LDH@FeNi_2_S_4_ core-shell nanocomposite [57]. In another work, a single-step solvothermal approach (ethylene glycol was used as the solvent) was reported for the Ni-Mn-S/RGO composite [58]. MOF-derived NMS materials have also been reported for SC applications. In this aspect, Cui et al. demonstrated the synthesis of RGO/Ni_2_ZnS_4_ composite using ZIF-8 through the Ni salt etching, high-temperature carbonization, and consequent hydrothermal vulcanization processes [59]. Furthermore, NiZn_2_O_4_/NiZn_2_S_4_ mesoporous nanocomposite was synthesized through a hydrothermal process on the NF substrate [60]. Overall, it can be concluded that several NS and NMS materials and their related composites have been investigated as energy storage/conversion electrodes. However, most of the synthetic approaches are based on hydrothermal treatments.

## 4. Energy Storage and Conversion Application of NS and NMS Materials

### 4.1. Supercapacitor (SC)

**Figure 6:**
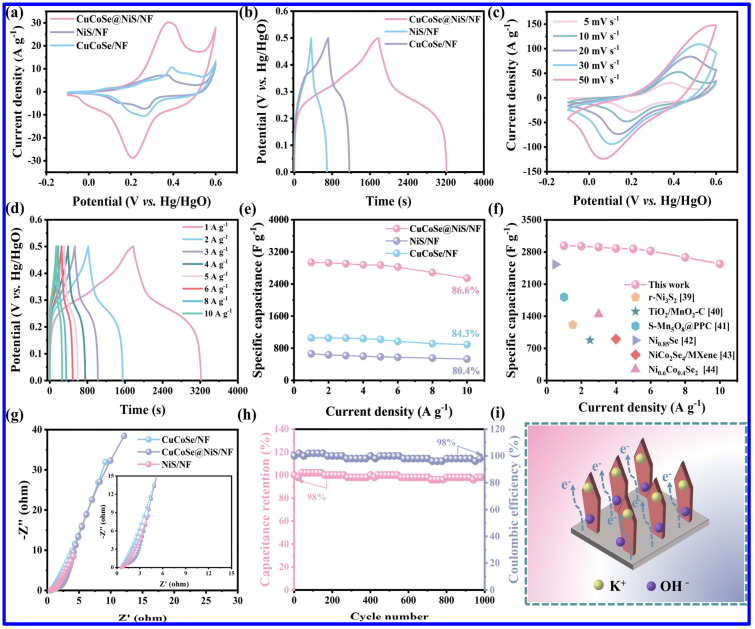
The SC performance of CuCoSe/NiS/NF composite and related electrode materials: (**a**) CV curves at 5 mV s^−1^, (**b**) GCD profiles at 1 A g^−1^ of NiS/NF, CuCoSe/NF and CuCoSe/NiS/NF; (**c**) CV curves and (**d**) GCD profiles of CuCoSe@NiS/NF; (**e**) Csp at different CDs for all electrodes; (**f**) comparison of the Csp of CuCoSe/NiS/NF and some related, reported electrodes; (**g**) Nyquist plots of all electrodes; (**h**) the CS curve of CuCoSe/NiS/NF, measured at 15 A g^−1^; (**i**) schematic representation of the electron and ion transport of CuCoSe/NiS/NF electrode during the charge–discharge process [63].

NS and NMS materials have been extensively utilized for the fabrication of SC electrodes in the last few years. It is important to note that NS materials exist in different phases, including α-NiS, β-NiS, NiS_2_, Ni_3_S_4_, Ni_7_S_6_, and Ni_3_S_2_. On the other hand, NS materials with diverse morphologies, including nanosheets, nanospheres, nanorods, etc., have been reported as the SC electrodes. For example, Yan et al. reported the evaluation of NiS nanosheets, grown on NF, as the SC electrode with a good specific capacitance (Csp) of 2587 F g^−1^ at a current density (CD) of 0.2 A g^−1^ [39]. Notably, the binder-less electrode further displayed a cycling stability (CS) of 95.8% after 4000 cycles. In order to demonstrate the practical utilization of the electrode, a hybrid SC device was also fabricated with an energy density (ED) of 38 Wh kg^−1^. In another work, a ternary composite based on NiS_1.03_, Ni_7_S_6_, and carbon exhibited the Csp of 1554.6 F g^−1^ [61]. Interestingly, the asymmetric device based on the ternary composite as the positive and the activated carbon as the negative electrode revealed relatively higher ED (41.2 Wh kg^−1^) than the above-discussed NiS/NF-based device. Additionally, the device also demonstrated a good CS of 86.8% after 10,000 cycles. In another study, the integration of NiS with functionalized MWCNTs enhanced the Csp from 1400 to 1966 F/g at a current density (CD) of 1 A g^−1^ [62]. Notably, the rate capability also enhanced from 64% to 78%. Such a significant increment in performance was credited to the increment in electrochemically active sites with the addition of MWCNT to the NiS. On the other hand, the synergy between the pseudocapacitive NS and EDLC-type CNT also played a significant role in improving the performance. The corresponding asymmetric device NiS/MWCNT//AC also displayed higher ED (74.1 Wh kg^−1^) than NiS//AC device (58 Wh kg^−1^). In another work, a binder-free SC electrode based on CuCoSe and NiS was grown on NF with an ultrathin nanosheet morphology [63]. Due to its high surface area and enhanced electrochemically active sites, the electrode exhibited better performance than its primary counterparts, such as NiS/NF and CuCoSe/NF. As shown in Figure 6a,b, the composite electrode displayed higher current response in CV curves and superior charge–discharge time in GCD profiles than NiS/NF and CuCoSe/NF. The CV and GCD profiles of CuCoSe/NiS/NF composite electrode demonstrate the sign of pseudocapacitive nature (Figure 6c,d). On the other hand, the composite also displayed a superior rate performance than its other counterparts and higher Csp than the other related, reported electrode materials, indicating its promising application potential in SCs (Figure 6e,f). Apart from these, the composite also displayed lower solution resistance and charge transfer resistance, signifying a better conductive nature (Figure 6g). Furthermore, the electrode retained 98% of its initial Csp over 1000 charge/discharge cycles (Figure 6h). As schematically shown in Figure 6i, the transport of electrons and electrolyte ions is found to be easier for the composite electrode than its related counterparts. As a whole, the electrode revealed the Csp of 2937.5 F g^−1^, and the corresponding SC device displayed a maximum ED of 41.8 Wh kg^−1^. The uniform nanosheet structures of the electrode provided adequate channels for the transport of electrolyte ions and also improved the surface area, improving the supercapacitive performance.

The NS materials have also been combined with EDLC-type 2D graphene materials. For example, Darsara et al. described the synthesis of NiS, Ni_3_S_4_, and RGO, which exhibited the Csp of 1578 F g^−1^ at the CD of 0.5 A g^−1^ in 2M KOH electrolyte [64]. The starfish-like 3D electrode also displayed a good CS of 91% after 5000 cycles. The electrochemical reactions of the NS materials during the charge–discharge process are shown below:

NiS + 3OH^−^ ↔ NiS(OH)_3_ + 3e^−^ (10)

Ni_3_S_4_ + OH^−^ ↔ Ni_3_S_4_OH + e^−^ (11)

**Figure 7:**
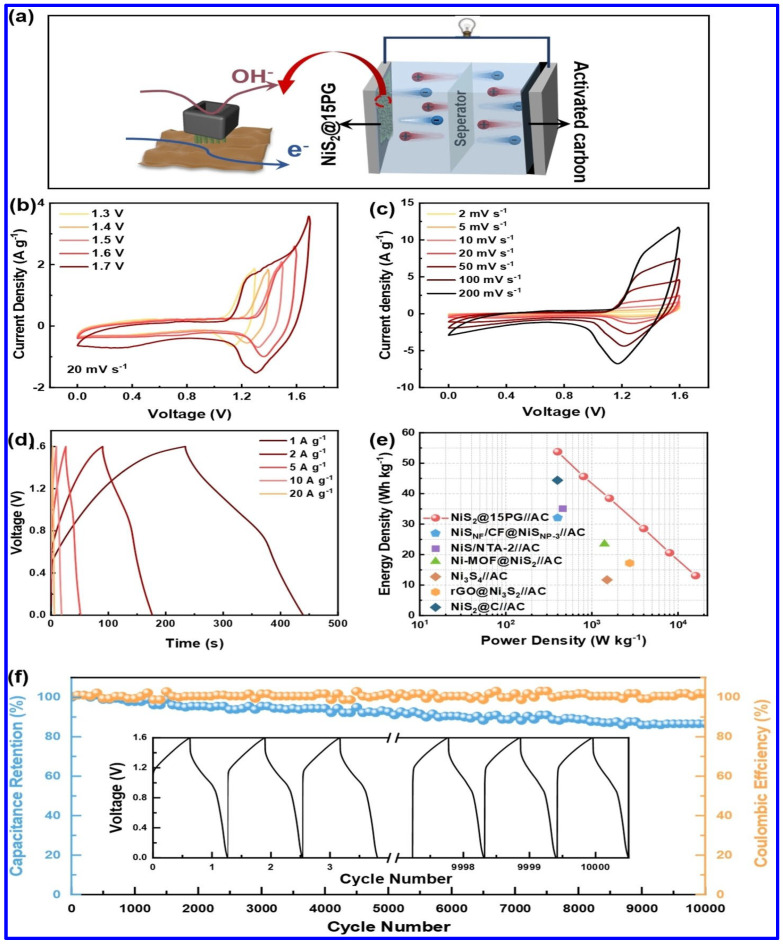
The electrochemical performance of NiS_2_/PANI/graphene oxide composite (NiS_2_@15PG)-based hybrid SC device: (**a**) Schematics of the device based on NiS_2_@15PG as positive and AC as negative electrode, (**b**) CV profiles at different voltages (20 mV s^−1^), ranging from 1.3 to 1.7 V, (**c**) CV curves at different scan rates (SRs), (**d**) GCD profiles at different CDs, (**e**) the Ragone plot, (**f**) the CS curve [65].

In another work, a hollow-structured composite electrode based on NiS_2_, PANI, and graphene oxide was reported to display a specific capacity (Cs) of 536.13 C g^−1^ at a CD of 1 A g^−1^ [65]. Notably, a hybrid SC device was fabricated in this work, using the composite as the positive and AC as the negative electrode. The schematics of the device are shown in Figure 7a. As shown in Figure 7b,c, the device displayed its potential to be operated at a high operating potential of 1.6 V. The GCD profiles demonstrate the superior redox behavior of the device (Figure 7d). Additionally, the device exhibited a maximum ED of 13.09 Wh kg^−1^, as observed from the Ragone plot in Figure 7e. Furthermore, the capacity retention of the device was found to be 86.59% over 10,000 charge/discharge cycles (Figure 7f).

It is evident that the NiS displayed higher electrochemical performance than its oxide counterpart. In this aspect, a recent article is dedicated to a direct comparison of the supercapacitive performance of such materials [66]. At the current density of 5 A g^−1^, NiS exhibited the Csp of 1066 F g^−1^, which was higher than NiO (Csp of 166 F g^−1^). The enhanced charge storage of the sulfide was attributed to its higher electrical conductivity, enhanced pseudocapacitive behavior, larger surface area, enhanced redox kinetics, and strong interaction with the electrolyte (3 M KOH).

**Figure 8:**
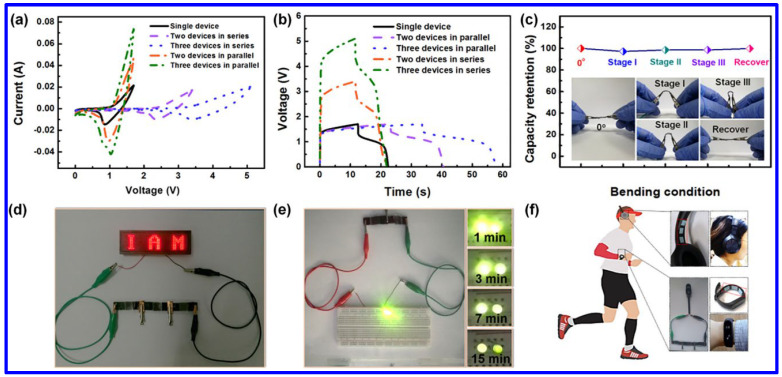
The electrochemical performance of the flexible NiCo_2_S_4_/Ni(OH)_2_//Fe_2_O_3_ device: the (**a**) CV and (**b**) GCD curves of the devices connected in series or in parallel at SR of 20 mV s^−1^ and CD of 50 mA cm^−2^, respectively; (**c**) the plot of capacity retention of the device at different bending angles, demonstrating superior flexibility, (**d**) the digital images of three flexible devices, connected in series, lighting red LED display, (**e**) the image of the glow up of yellow LED with the series connection of two flexible devices, and (**f**) the image of three in series connection for functioning a headphone and an electronic watch [56].

Among the NMS materials, NiCo_2_S_4_ is the most-studied one because of its easy synthetic approaches, high chemical stability, and enhanced redox characteristics. Chen et al. revealed the fabrication of a flexible SC electrode by integrating NiCo_2_S_4_ with Ni(OH)_2_ [56]. The mesh-like electrode displayed an areal capacity of 246.9 mAh g^−1^. Additionally, the solid-state asymmetric SC (ASC) device exhibited the ED of 315 μWh cm^−2^ at the corresponding power density of 2.14 mW cm^−2^. Additionally, the operating voltage of the device has been enhanced to 5V by connecting three devices in series. Furthermore, the current response could be improved by connecting three devices in parallel, as demonstrated in Figure 8a,b. Negligible loss of capacity has been monitored at various bending angles of the device, indicating its promising flexibility (Figure 8c). The practical utility of the devices was further confirmed by glowing red and green LEDs after charging the device for a definite time interval (Figure 8d,e). Notably, the advanced SC device in connection with another unit was able to power a headphone and an electronic watch, enabling its significant potential for future electronics (Figure 8f). In another work, the hydrothermally synthesized Ni_2_ZnS_4_/RGO composite exhibited the Csp of 1150 F g^−1^ at the CD of 1 A g^−1^ [59]. However, the CS of the composite was found to be poor (capacitance retention of 59.7% after 2000 cycles), which can be attributed to the structural breakdown during the repeated charge/discharge process. The Csp was further enhanced by combining RGO with Ni-Mn-S. The solvothermally synthesized composite electrode displayed a superior Csp of 2042.22 F g^−1^ at the CD of 1 A g^−1^ [58]. Compared to the previously discussed Ni_2_ZnS_4_/RGO composite, the electrode also demonstrated better CS of 77.78% over 5000 cycles. Additionally, the SC device based on the composite electrode as the positive and AC as the negative electrode retained 81.97% of its initial capacitance over 10,000 charge–discharge cycles. In another work, the hydrothermally synthesized Ni_1-x_Cu_x_S displayed a maximum Csp of 1092 F g^−1^, which was found to be higher than its single metal sulfide counterparts like NiS (575 F g^−1^) and Cu_9_S_5_ (70 F g^−1^) [67]. This study signifies the importance of MNS materials in the field of energy storage. Alitabar et al. reported the hydrothermal growth of Ni-Co-Fe-S nanosheets on NF, which exhibited the Cs of 1156 C g^−1^ and the rate capability of 81.1% [68]. The enhanced electrochemical performance of the battery-type SC electrode was ascribed to its high surface area (84.3 m^2^ g^−1^) and enhanced porosity. The superior redox activity was identified from the noticeable redox peaks in CV curves at different SRs, which can be assigned to the following redox reactions.

Ni_3_S_2_ + 3OH^−^ ↔ Ni_3_S_2_(OH)_3_ + 3e^−^ (12)

FeS_2_ + OH^−^ ↔ FeS_2_OH + e^−^ (13)

CoS_2_ + OH^−^ ↔ CoS_2_OH + H_2_O + e^−^ (14)

CoS_2_OH + OH^−^ ↔ CoS_2_O + H_2_O + e^−^ (15)

In another work, a core-shell structured composite electrode based on FeNi_2_-LDH and FeNi_2_S_4_ was reported to deliver a Cs of 450 C g^−1^ at a high CD of 6 A g^−1^ [57]. Notably, the electrode displayed a good CS of 92.3% over 5000 cycles due to its enhanced structural stability and higher porosity. Table 1 represents a comparative study on the electrochemical performance of a few NS and MNS materials. The electrochemical performance of these electrodes was reported to be measured in aqueous KOH electrolyte. However, the concentration of the electrolyte varies in different works. As shown in the table, the sulfide-based electrodes have been combined with other metal sulfides, oxides, carbon materials, LDH, etc. The electrode demonstrated good charge storage performance in terms of superior capacitance/capacity and CS over longer charge/discharge cycles. Jinlong et al. compared the electrochemical performance of NF-grown NiCo_2_O_4_ and NiCo_2_S_4_ [69]. The NMS material exhibited a higher surface area (75.8 m^2^ g^−1^) than its oxide counterpart (41.84 m^2^ g^−1^). Accordingly, the mixed sulfide also displayed better cycling stability (92.1%) than the mixed oxide (84.8%) over 5000 charge–discharge cycles.

Overall, the enhanced charge storage of the Ni-based electrodes is ascribed to their improved redox activity, enhanced porosity, and high surface area. Benefited by such characteristics, Ni-based materials have also been employed for energy conversion applications. The next section is dedicated to exploring the energy conversion (OER) applications of such materials.

### 4.2. OER

**Figure 9:**
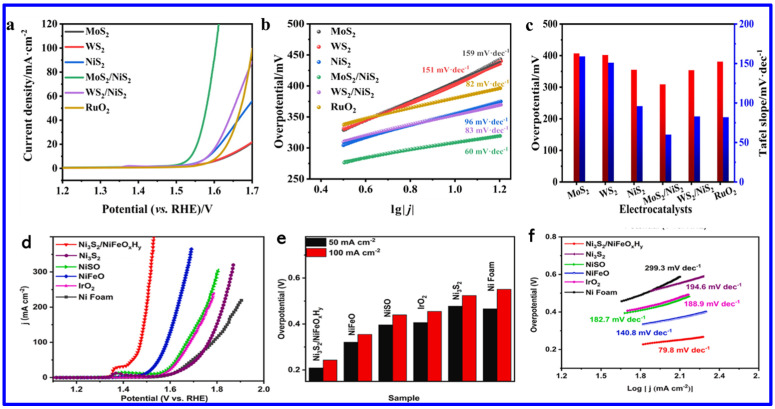
OER activity of various NS materials: (**a**) LSV curves, (**b**) Tafel slopes, and (**c**) comparison of OPs at 10 mA cm^−2^ and Tafel slope for MoS_2_, WS_2_, NiS_2_, MoS_2_/NiS_2_, WS_2_/NiS_2_, and RuO_2 2_ [77]; (**d**) OER LSV curves, (**e**) OPs at 50 and 100 mA cm^−1^, and (**f**) Tafel plots for NF, IrO_2_, NiSO, NiFeO, Ni_3_S_2_, and Ni_3_S_2_/NiFeO_x_H_y_ [78].

Among the various transition metal sulfides/mixed sulfides, NS and NMS have emerged as one of the most effective electrocatalysts for water splitting, particularly for the oxygen evolution reaction (OER). Specifically, NS’s exceptional performance is attributed to several factors: it exists in multiple crystalline phases (such as NiS, NiS_2_, and Ni_3_S_2_), many of which exhibit low OPs, enhancing catalytic efficiency; it shows excellent electrochemical stability in alkaline media, which is essential for long-term durability; and its polymorphic forms and ability to form heterostructures contribute to a high density of active sites, thereby improving catalytic activity. Furthermore, nickel is significantly more abundant and cost-effective compared to precious metals like platinum, ruthenium, and iridium, making nickel sulfide a practical and scalable alternative for OER through electrochemical water splitting. An excellent electrocatalyst for OER should possess key characteristics such as low OP, fast reaction kinetics, a large electrochemically active surface area (ECSA), low charge transfer resistance (Rct), and high long-term stability. Recently, various synthesis methods have been employed to optimize these parameters in NS materials, aiming to significantly enhance their OER performance. For instance, Ni et al. employed a straightforward solid-state synthesis method to fabricate transition-metal sulfide heterostructures, specifically MoS_2_/NiS_2_ and WS_2_/NiS_2_ [77]. These heterostructures exhibited significantly improved OER performance, with low OPs of 300 mV for MoS_2_/NiS_2_ and 320 mV for WS_2_/NiS_2_ at a CD of 10 mA cm^−2^. Additionally, they achieved smaller Tafel slopes of 60 mV dec^−1^ and 83 mV dec^−1^, respectively, in 1 M KOH solution. These results not only surpass the performance of their individual components, MoS_2_, WS_2_, and NiS_2_, but also outperform the benchmark RuO_2_ catalyst, highlighting the potential of heterostructure engineering as a powerful strategy for advancing NS-based electrocatalysts (Figure 9a–c) [77].

**Figure 10:**
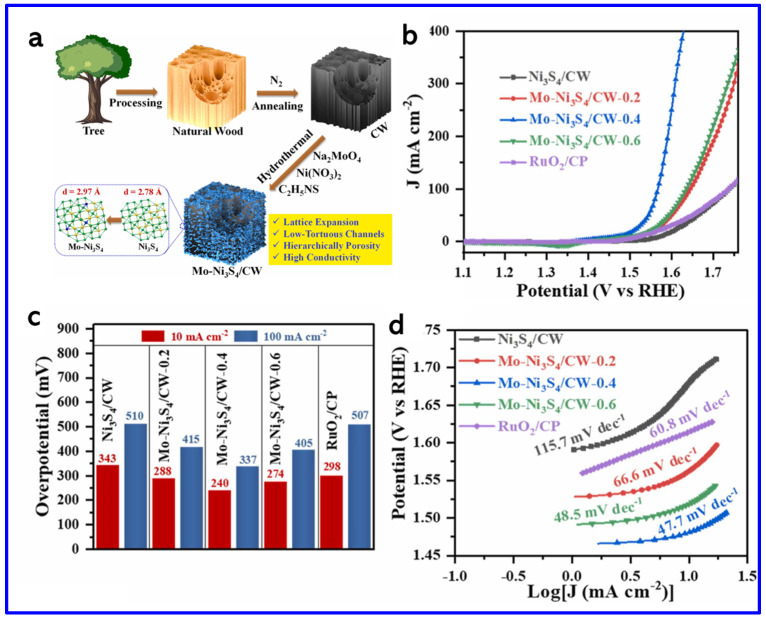
(**a**) Schematics of the synthesis of Mo-Ni_3_S_4_/CW, (**b**) the LSV curves, (**c**) corresponding OP histograms at 10 and 100 mA cm^−2^, and (**d**) Tafel plots [79].

In another work, Zhou et al. synthesized a Ni_3_S_2_/NiFe-(oxy) hydroxide (NiFeO_x_H_y_) heterostructure nanosheet array that exhibited outstanding OER performance with ultralow OPs of 209 mV and 243 mV to achieve the current densities (CDs) of 50 and 100 mA cm^−2^, respectively (Figure 9d–f). These values are found to be lower than those of Ni_3_S_2_ (480 mV and 534 mV), NiSO (397 mV and 440 mV), NiFeO (321 mV and 355 mV), commercial IrO_2_ (406 mV and 455 mV), and NF (466 mV and 551 mV), demonstrating superior catalytic activity. Notably, Ni_3_S_2_/NiFeO_x_H_y_ outperformed even the commercial IrO_2_ catalyst. The Tafel slope of Ni_3_S_2_/NiFeO_x_H_y_ was 79.8 mV dec^−1^ at high CD (~100 mA cm^−2^), which is much smaller than those of Ni_3_S_2_ (194.6 mV dec^−1^), NiSO (182.7 mV dec^−1^), NiFeO (140.8 mV dec^−1^), IrO_2_ (188.9 mV dec^−1^), and NF (299.3 mV dec^−1^), indicating faster OER kinetics (Figure 9f). This remarkable activity arose from the synergistic coupling between Ni_3_S_2_ and NiFeO_x_H_y_ phases, which enhanced charge transfer, increased the density of active sites, and improved overall stability [78]. On the other hand, Shi et al. synthesized molybdenum-doped Ni_3_S_4_ nanosheets grown on carbonized wood (Mo-Ni_3_S_4_/CW) [79]. The synthetic approach is schematically shown in Figure 10a. The theoretical calculations revealed that Mo-doping caused lattice expansion in Ni_3_S_4_, which optimized the adsorption energies of hydrogen and oxygen species. This adjustment regulated the local charge density at active sites, thereby enhancing the OER activity (Figure 10b). The Mo-Ni_3_S_4_/CW-0.4 catalyst exhibited OPs of 240 mV and 337 mV to reach CDs of 10 and 100 mA cm^−2^, respectively, outstripping undoped Ni_3_S_4_/CW (η_10_ = 343 mV) (Figure 10c). Moreover, Mo-Ni_3_S_4_/CW-0.4 showed the lowest Tafel slope of 47.7 mV dec^−1^, indicating superior OER kinetics compared to Ni_3_S_4_/CW (115.7 mV dec^−1^) and even commercial RuO_2_ (66.6 mV dec^−1^) (Figure 10d).

**Figure 11:**
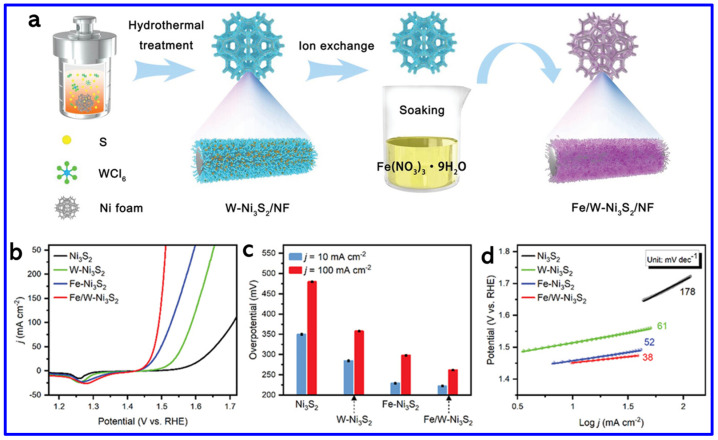
(**a**) Schematics of the synthesis process of Fe/W-Ni_3_S_2_, (**b**) LSV curves for OER of Ni_3_S_2_, W-Ni_3_S_2_, Fe-Ni_3_S_2_, and Fe/W-Ni_3_S_2_ (with 85% iR-compensation). (**c**) OPs at different CDs. (**d**) Corresponding Tafel slopes [81].

Metal-doping is another strategy for enhancing the catalytic activity of NS materials. In this context, Prakash et al. synthesized Zn-doped NiS nanosheets on NF using a simple hydrothermal method [80]. The optimized sample, Zn-NiS-3, exhibited the best OER performance, achieving an OP of 320 mV at a CD of 50 mA cm^−2^, which is significantly lower than that of undoped NiS. The enhanced catalytic activity is attributed to the incorporation of Zn, which likely improves the electronic structure and promotes charge transfer during the oxygen evolution reaction. In another work, Wang et al. developed a dual-doped NS catalyst (Fe/W-Ni_3_S_2_) grown on NF, exhibiting significantly enhanced OER performance [81]. The hydrothermal-assisted ion-exchange process was adopted to grow the doped NS material on NF, as shown in Figure 11a. The improved activity is attributed to the synergistic effect between iron doping and nickel vacancies introduced by tungsten leaching during the in situ oxidation process. This combination maximizes the exposure of active OER sites and enhances charge transport, thereby accelerating the reaction kinetics (Figure 11b). The Fe/W-Ni_3_S_2_ catalyst achieved low OPs of 222 mV and 261 mV at CDs of 10 and 100 mA cm^−2^, respectively, outperforming Fe-Ni_3_S_2_ (229 and 298 mV), W-Ni_3_S_2_ (284 and 358 mV), and undoped Ni_3_S_2_ (350 and 479 mV) (Figure 11c). It also exhibited the lowest Tafel slope of 38 mV dec^−1^, compared to 52, 61, and 178 mV dec^−1^ for Fe-Ni_3_S_2_, W-Ni_3_S_2_, and Ni_3_S_2_, respectively, confirming its enhanced OER kinetics (Figure 11d). These findings demonstrate that dual doping is an effective strategy for boosting the catalytic performance of NS materials.

A recent work reported the synthesis of Cu and Co co-doped Ni_3_S_2_ catalyst (CuCo-Ni_3_S_2_/NF) on NF using a hydrothermal method followed by liquid-phase vulcanization [82]. The optimized catalyst, with a 1:1 molar ratio of copper to cobalt, exhibited higher OER performance, achieving a low OP of 400 mV at a CD of 100 mA cm^−2^. This outperforms Co-Ni_3_S_2_/NF (425 mV), Cu-Ni_3_S_2_/NF (430 mV), Ni_3_S_2_/NF (438 mV), and even RuO_2_/NF (460 mV) under the same conditions. The improved catalytic activity is attributed to the synergistic effects among the three transition metals. Specifically, Co-doping increased the number of exposed active sites, while Cu incorporation enhanced the charge transfer rate. Together, these factors contributed to the superior electrocatalytic performance of CuCo-Ni_3_S_2_/NF. Furthermore, Mao et al. prepared Fe and Co dual-doped Ni_3_S_4_ nanosheets using a two-step hydrothermal method [83]. The dual-doped catalyst demonstrated excellent OER performance, with OPs of 230 mV and 280 mV at CDs of 10 and 100 mA cm^−2^, respectively. These values are significantly lower than those for Fe-Ni_3_S_4_ (257 and 307 mV), Co-Ni_3_S_4_ (260 and 317 mV), pure Ni_3_S_4_ (268 and 357 mV), and the precursor nanosheets (238 and 310 mV). The improved performance is attributed to a combination of high Ni^3+^ content that enhances OH^−^ adsorption and electron transfer, strong adhesion to the NF substrate for improved conductivity and stability, and synergistic effects from dual cation doping. Additionally, oxygen doping or surface hydroxides formed during OER may further boost catalytic activity.

The morphology and crystal phase have a significant impact on the OER efficiency of NS materials. In this aspect, Manjunatha et al. synthesized various polymorphs of nickel sulfide via a hydrothermal method and investigated, for the first time, the influence of morphology and crystalline structure on OER performance [84]. The catalytic activity followed the order: NiS > NiS_2_ > Ni_3_S_4_. Among the different morphologies, NiS (sugar-cube-shaped) demonstrated the best performance, followed by NiS (agglomerated stone particles) and NiS (apple-shaped). For NiS_2_, the order was: aggregated stone particles > rose-type > tubular bacteria-shaped. Notably, NiS (sugar cubes) achieved an OPof 362 mV at 20 mA cm^−2^, outperforming even the benchmark RuO_2_, which showed an OPof 416 mV under the same conditions. In another work, the NiS/rGO nanocomposite demonstrated a markedly higher CD (1367 mA cm^−2^) compared to pure NiS (536 mA cm^−2^) and rGO (115 mA cm^−2^), owing to the strong chemical interaction between nickel sulfide and the rGO nanosheets [85]. Additionally, the nanocomposite exhibited a lower onset potential of 1.40 V, indicating a reduced energy barrier for initiating the OER activity. According to the Tafel plots, the NiS/rGO composite showed the smallest Tafel slope (32 mV dec^−1^), outperforming both NiS (52 mV dec^−1^) and rGO (46 mV/dec), further revealing OPs of 216 mV for NiS, 369 mV for rGO, and only 162 mV for the composite at 10 mA cm^−2^. The hetero-atom doping of carbon materials, in combination with NS materials, displayed better catalytic activity than their undoped counterparts. In a recent work, it has been observed that the N-doped graphene-like carbon layers/Ni_3_S_2_ composite, grown on NF, exhibited better OER activity compared to graphene-like carbon nanosheets/Ni_3_S_2_ composite [86]. This enhanced performance is attributed to the unique heterogeneous architecture formed by N-doped CNT and Ni_3_S_2_ nanowires, which synergistically improved catalytic efficiency. In another work, the NiS/NiO/N-doped CNT, grown on NF, demonstrated superior OER performance, achieving a notably low OPof 269 mV at 10 mA cm^−2^ [87]. This value is significantly lower than those recorded for NiO/N-doped CNT/NFs (390 mV), Ni/N-doped CNT/NF (470 mV), and commercial RuO_2_ (347 mV). The Tafel slope analysis further confirmed the enhanced kinetics, with NiS/NiO/N-doped CNT/NFs showing a much smaller slope of 48.4 mV dec^−1^ compared to 221.4, 140.5, and 67.1 mV dec^−1^ for NiO/N-doped CNT/NFs, Ni/N-doped CNT/NFs, and RuO_2_, respectively. These findings suggest that the formation of NiS/NiO heterostructures significantly boosts both catalytic efficiency and reaction kinetics in OER. NMS materials have also been combined with other advanced functional materials for the fabrication of electrocatalysts for OER applications. In this aspect, Yang et al. reported the fabrication of an OER catalyst based on 3D heterostructures by adhering needle-like NiCo_2_S_4_ particles on NiCo alloys embedded N-doped carbon fibers [88]. Owing to the synergistic contribution from the alloy and the semiconductor-type carbon fibers, the heterostructured composite achieved a low OP of 291 mV at the CD of 10 mA cm^−2^. Even at a higher CD of 100 mA cm^−2^, the composite electrode exhibited an OP of 376 mV. In another work, the addition of Au significantly improved the catalytic activity of NiCo_2_S_4_ [89]. Compared to the base NMS materials, the composite electrode (312 mV) displayed a lower OP of 299 mV. Notably, the Tafel slope has also been reduced in the composite (44.5 mV dec^−1^), in comparison with the bare NMS material (49.1 mV dec^−1^). The NMS material was further combined with carbon materials. For instance, a recent work demonstrated the catalytic activity of a composite based on NiCo_2_S_4_ and S-doped g-C_3_N_4_ [90]. Benefitting from the high surface area and strong synergy between the individual components, the composite demonstrated a lower OP of 370 mV. It is important to note that the stability of NMS materials depends on several factors, including the stoichiometry, composition, chemical structure, and electrolyte, as well as surface transformation during OER activity. In some cases, the actual active species for OER could be the oxidation product of Ni-based sulfides, like Ni-based (oxy)hydroxides, formed through the surface transformation under OER conditions.

Apart from the NiCo_2_S_4_, other NMS materials have also been reported for enhanced OER activities. In this aspect, Ke et al. reported the fabrication of MOF-derived chalcogenide electrocatalysts based on Fe_0.75_Ni_0.25_S_2_ and FeNiOOH [91]. The composite exhibited a lower OP of 247 mV, along with a smaller Tafel slope of 47.6 mV dec^−1^. In another work, the electrocatalyst based on Ni_1.29_Co_1.49_Mn_0.22_S_4_ displayed good OER activity with a low OP of 348 mV at the CD of 10 mA cm^−2^ [92]. The catalyst also exhibited good activity towards the oxygen reduction reaction, indicating its potential applicability in Zn-air batteries. The enhanced catalytic performance was attributed to the high surface area, favorable electronic structure, and strong synergy between the various metal dopant species. Yang et al. reported the formation of the heterostructures of Ni_3_S_2_ and FeNi_2_S_4_ on NF. The Ni form grown electrocatalyst exhibited an OP of 235 mV at the CD of 10 mA cm^−2^ [93]. In another work, the NF-grown Ni_3_S_2_/Co_3_S_4_ nanosheets demonstrated promising OER activity in alkaline seawater [94]. Such a seawater electrocatalysis process was found to be stabilized at higher current density up to 800 mA cm^−2^. Furthermore, powered by a solar cell, the integrated device exhibited advanced seawater splitting with a high solar-to-hydrogen efficiency of 15.13%. These studies clearly demonstrate the promising electrocatalytic activities of NS and NMS materials. A comparative study on the OER activity of a few NS and NMS materials is summarized in Table 2. As shown in Table 2, the electrocatalysts exhibited lower OP and Tafel slope.

## 5. Conclusions and Future Prospects

Ni-based chalcogenides have been efficiently integrated with other functional materials for high-performance energy storage and conversion applications in the last few years. Their enhanced characteristics, like high surface area, enhanced porosity, easy synthetic approaches, rich redox activity, and high abundance, permitted them to serve as effective electrode materials for SC and OER applications. Recent advancements of these materials and their electrochemical applications are systematically summarized in this review. Despite several advantages, a few drawbacks of these materials hamper their effective utilization in the field of energy storage and conversion. The existing challenges and the probable solutions are briefly summarized below:

1. The detailed charge storage mechanism of NMS materials has not been clearly explored yet. In this aspect, the suitable integration of theoretical calculations and experimental results is highly essential.
2. The subsequent volume expansion and the fallout of the active SC electrode materials from the current collectors negatively affect the cycling performance and rate capability. To solve this issue, specifically designed electrode materials with high chemical stability are essential.
3. All the relevant works reported the evaluation of the electrochemical performance of NS and NMS materials in KOH electrolyte. Further increment of electrochemical performance is possible through the addition of redox species in the electrolyte. However, in those cases, the cycling performance of the electrodes should be monitored carefully.
4. The charge storage kinetics should also be evaluated thoroughly. In the case of OER activity, the NMS materials have the tendency to oxidize easily to their oxide and hydroxide counterparts, which sometimes harms the OER activities by deteriorating stability and catalytic activity. To solve this issue, the NMS materials have been combined with metal oxides and hydroxides and sometimes grown on conductive substrates. However, in those cases, the catalyst loading should be provided with accuracy.
5. The in situ characterization techniques should be implemented to understand several factors, like the charge storage mechanism, the sources of enhanced catalytic activity, the interaction between the electrode and electrolytes, intermolecular interactions, and structural changes of the electrode during the repetitive electrochemical processes.

Witnessing the rapid progress in such materials in various fields, it is expected that the existing issues with the energy storage and conversion applications of NS and NMS materials will be systematically solved in the upcoming years.

## Figures and Tables

**Table 1:** Comparison of the electrochemical performance of a few NS and NMS materials for SC applications.

Electrode	Electrolyte (Conc. of KOH)	Specific Capacitance (F g^−1^) or Specific Capacity (C g^−1^)	Cycling Stability (%)	Ref.
β-NiS	2 M	638.34 C g^−1^ at 1 A g^−1^	-	[40]
NiS/NF	3 M	2587 F g^−1^ at 0.2 A g^−1^	95.8 after 4000 cycles	[39]
α-NiS/β-NiS	3 M	2250 F g^−1^ at 2 mV s^−1^	-	[47]
NiS_1.03_/Ni_7_S_6_/Carbon	6 M	1554.6 F g^−1^ at 1 A g^−1^	80.4 after 5000 cycles	[61]
NiS/functionalized-MWCNT	6 M	1966 F g^−1^ at 1 A g^−1^	86.2 after 10,000 cycles	[62]
Co_3_O_4_/NiS/NF	6 M	1395.3 F g^−1^ at 1 A g^−1^	89.9 after 5000 cycles	[70]
CuCoSe/NiS/NF	3 M	2937.6 F g^−1^ at 1 A g^−1^	98 after 1000 cycles	[63]
Ni_3_S_4_/NiS/RGO	2 M	1578 F g^−1^ at 0.5 A g^−1^	91 after 5000 cycles	[64]
NiS_2_/PANI/graphene	1 M	536.13 C g^−1^ at 1 A g^−1^	80.5 after 5000 cycles	[65]
P-doped Co_3_S_4_/Ni_3_S_4_/NF	2 M	3614 F g^−1^ at 1 A g^−1^	73 after 3000 cycles	[43]
Ni_3_S_2_/CoMoS_4_/MnO_2_/NF	1 M	2021 F g^−1^ at 1 A g^−1^	90 after 4000 cycles	[71]
Mg(OH)_2_/Ni_3_S_4_	1 M	3316.7 F g^−1^ at 1 A g^−1^	-	[49]
Cu2S/NiS/Ni_3_S_4_	6 M	363.05 mAh g^−1^ at 0.5 A g^−1^	94.8 after 8000 cycles	[42]
Co_9_S_8_/NiS_2_/Cu_2_S/NF	3 M	460.15 C g^−1^ at 1 A g^−1^	-	[48]
NiCo_2_S_4_	3 M	3506 F g^−1^ at 2 A g^−1^	90 after 5000 cycles	[51]
NiCo_2_S_4_/functionalized MWCNT	6 M	1360 F g^−1^ at 1 A g^−1^	80.6 after 10,000 cycles	[52]
NiCo_2_S_4_/MoS_2_	3 M	2594 F g^−1^ at 0.8 A g^−1^	192 after 45,000 cycles	[54]
NiCo_2_S_4_/SnS_2_	1 M	329.22 mAh g^−1^ at 2 A g^−1^	76.87 after 10,000 cycles	[72]
NiCo_2_S_4_/CoAl-LDH	3 M	2120 F g^−1^ at 1 A g^−1^	98.96 after 10,000 cycles	[73]
NiCo_2_S_4_/Mo-doped Co-LDH/carbon cloth	3 M	3049.3 F g^−1^ at 1 A g^−1^	91 after 10,000 cycles	[74]
FeNi_2_-LDH/FeNi_2_S_4_	1 M	806 C g^−1^ at 1 A g^−1^	92.3 after 5000 cycles	[57]
Ni-Co-Mn-S	6 M	661 C g^−1^ at 1 A g^−1^	-	[75]
Zn-Ni-Co-S/Zn-Ni-Co-O	6 M	1445 C g^−1^ at 1 A g^−1^	86.1 after 3000 cycles	[76]
NiZn_2_O_4_/NiZn_2_S_4_/NF	1 M	1516 C g^−1^ at 1 A g^−1^	86.9 after 10,000 cycles	[60]
Ni_1.43_Fe_0.5_Co_0.5_S_0.97_/NF	6 M	1156 C g^−1^ at 2 A g^−1^	92.9 after 10,000 cycles	[68]
Ni_2_ZnS_4_/RGO	6 M	1150 F g^−1^ at 1 A g^−1^	59.7 after 2000 cycles	[59]
Ni-Mn-S/RGO	2 M	2042.22 F g^−1^ at 1 A g^−1^	77.78 after 5000 cycles	[58]

**Table 2:** Comparison of the electrochemical performance of a few NS and NMS materials for OER applications.

Sl. N.	Materials	Overpotential (mV) at 10 mA cm^−2^	Tafel Slope (mV dec^−1^)	Ref.
1.	NiCo_2_S_4_/Carbon Nitrogen nanosheets	360	76	[95]
2.	Zn-doped NiS	320 (at 50 mA cm^−2^)	36	[80]
3.	Ni_1.5_Co_1.5_S_4_	360	61	[96]
4.	Ni_1.29_Co_1.49_Mn_0.22_S_4_	348	65	[92]
5.	NiS	362 (at 20 mA cm^−2^)	65	[84]
6.	Co-Ni_3_S_2_/NF	274	199	[97]
7.	Fe/W-doped Ni_3_S_2_	222	38	[81]
8.	Fe_0.75_Ni_0.25_S_2_/FeNiOOH	247	47.6	[91]
9.	CuNiS	337	43	[98]
10.	NiS	210	60	[99]
11.	Ni_3_S_2_/CoS_x_	226	56	[100]
12.	NiS/rGO	162	32	[85]
13.	Ni_3_S_2_/FeNi_2_S_4_/NF	235	92	[93]
14.	FeCO/Ni_3_S_4_	230 (at 20 mA cm^−2^)	63.2	[83]
15.	V-doped Ni_3_S_2_/NF	268	99	[101]
16.	N-doped CNTs/NiS_2_	235	88	[102]
17.	Mo-Ni_3_S_4_/Carbonized wood	240	47.7	[79]
18.	P-doped Ni_3_S_2_/NiS/NF	178	37	[103]
19.	Co-doped Ni_3_S_2_/NF	297 (at 20 mA cm^−2^)	50.3	[104]
20.	NiCo_2_S_4_/NiCo alloys/N-doped carbon fibers	291	51.17	[88]
21.	Au/NiCo_2_S_4_	299	44.5	[89]
22.	NiCo_2_S_4_/S-doped g-C_3_N_4_	370	99.2	[90]

## Data Availability

No new data were created.
